# Metabolomic profiling reveals amino acid and carnitine alterations as metabolic signatures in psoriasis

**DOI:** 10.7150/thno.51154

**Published:** 2021-01-01

**Authors:** Chao Chen, Guixue Hou, Chunwei Zeng, Yan Ren, Xiang Chen, Cong Peng

**Affiliations:** 1 Department of Dermatology, Xiangya Hospital, Central South University, Changsha, Hunan, China; 2 Hunan Key Laboratory of Skin Cancer and Psoriasis, Xiangya Hospital, Central South University, Changsha, Hunan, China; 3 Hunan Engineering Research Center of Skin Health and Disease, Xiangya Hospital, Central South University, Changsha, Hunan, 410008, China; 4 BGI-Shenzhen, Shenzhen, Guangdong, 518083, China; 5 National Clinical Research Center for Geriatric Disorders, Xiangya Hospital, Central South University, Changsha, Hunan, 410008, China

**Keywords:** psoriasis, metabolomics, amino acid, L-carnitine, Th17

## Abstract

High-throughput metabolite profiling provides the opportunity to reveal metabolic mechanisms and identify biomarkers. Psoriasis is an immune-mediated chronic inflammatory disease. However, the role of metabolism in psoriasis pathogenesis remains unclear.

**Methods:** Plasma samples of individuals (45 psoriasis and 45 sex‐, age-, and BMI-matched healthy controls) were collected. Non-targeted metabolomics and amino acid- or carnitine-targeted metabolomics were conducted, then, plasma samples of mice induced by imiquimod (IMQ) were subjected to the amino acid- and carnitine-targeted metabolomic profiling. Flow cytometry was used to study the effect of L-carnitine (LC(C0)) on IMQ-induced psoriatic inflammation.

**Results:** Through the non-targeted metabolomics approach, we detected significantly altered amino acids and carnitines in psoriasis patients. Amino acid-targeted metabolomic profiling identified 37 amino acids altered in psoriasis, of these 23 were markedly upregulated, including essential amino acids (EAAs), and branched-chain amino acids (BCAAs), whereas glutamine, cysteine, and asparagine were significantly down-regulated. Carnitine-targeted metabolomic profiling identified 40 significantly altered carnitines, 14 of which included palmitoylcarnitine (C16) and were markedly downregulated in psoriasis, whereas hexanoylcarnitine (C6) and 3-OH-octadecenoylcarnitine (C18:1-OH) were significantly upregulated. Interestingly, glutamine, asparagine, and C16 levels were negatively correlated with the PASI score. Moreover, a higher abundance of LC(C0) was associated with markedly reduced IMQ-induced epidermal thickening and infiltration of Th17 cells in skin lesions, indicating LC(C0) supplementation as a potential therapy for psoriasis treatment.

**Conclusion:** Our results suggested the metabolism of amino acids and carnitines are significantly altered in psoriasis, especially the metabolism of EAAs, BCAAs, and LC(C0), which may play key roles in the pathogenesis of psoriasis.

## Introduction

Psoriasis is an immune cell-mediated inflammatory disease, and an imbalance of T cells is believed to play a key role in its pathogenesis 1. The IL-23/IL-17A-Th17 axis is one of the most critical pathways in psoriasis pathogenesis, coordinated by abundant pro-inflammatory cytokines and chemokines, including IL-17, TNF-α, IL-1β, and IL-6 1, 2. Evidence indicates that TNF-α and IL-1β facilitate insulin resistance development by elevating blood insulin and HDL cholesterol levels 3 and insulin resistance suppresses keratinocyte differentiation, resulting in persistent proliferation 4. Moreover, the IL-23/Th17 axis has the diabetogenic potential 5, and psoriasis has been identified as a risk factor for metabolic diseases due to shared pathogenic pathways, including the IL-23/Th17 axis 6-8.

Psoriasis is frequently associated with other systemic diseases such as obesity, diabetes or insulin resistance, and serum dyslipidemia 9. These metabolic disorders significantly increase psoriasis risk due to the chronic low‐grade inflammatory status and are associated with a worse prognosis 9, 10. For example, they could negatively affect the clinical response when patients with psoriasis receive biologics 11, 12. Interestingly, psoriasis patients have been reported to be more prone to developing insulin resistance than healthy individuals, indicating that psoriasis might be a metabolic disease to some extent 13.

Metabolomics is an emerging technique that analyzes the end-point products of metabolism 14-17. However, investigation of psoriasis by metabolomics has been limited. Nuclear magnetic resonance (NMR) spectroscopy was conducted to analyze metabolic profiles in psoriatic skin lesions, non-lesional skin, and psoriatic skin after corticosteroid treatment 18, The analysis showed that metabolites, such as glucose and myo-inositol, were significantly altered in psoriasis patients compared with the non-lesional skin, and, after treatment, these metabolite levels were returned to normal 18. The non-targeted metabolomics (LC-MS) was also performed to measure metabolite alterations in severe psoriasis after anti-TNF-α treatment. Various amino acids, such as proline, arginine, glycine, threonine, and aspartate, were closely associated with after anti-TNF-α treatment in psoriasis patients 19.

L-carnitine (LC), also known as free carnitine (C0), is considered a conditionally essential nutrient that plays a functional role in energy metabolism and fatty acid oxidation 20. Recently, carnitine deficiency has been widely reported in disorders such as diabetes, cancer, malnutrition, sepsis, aging, and psoriasis 21, 22. For example, LC(C0) can serve as a treatment for childhood psoriatic onycho-pachydermo-periostitis (POPP) 23, and clinical improvement in psoriatic arthritis symptoms has been detected during treatment for infertility with LC(C0) 24. These two observations indicated a potential therapeutic property of LC(C0) for psoriasis. Furthermore, carnitine has been shown to have important anti-inflammatory, antioxidant, lipid-lowering, and organ-supporting properties 20, 22, 25. However, the specific role of carnitine metabolism in the pathogenesis of psoriasis remains unclear.

In this study, we conducted plasma non-targeted and targeted metabolomic profiling in psoriasis patients and IMQ-induced psoriasis-like mouse model to explore the role of metabolism in psoriasis. The findings show that the metabolism of amino acids and carnitines is significantly altered in psoriasis, especially of EAAs and BCAAs, suggesting that this altered metabolism may play a key role in the pathogenesis of psoriasis. Most importantly, we identified the protective roles of LC(C0) in the pathogenesis of psoriasis, providing a novel strategy for psoriasis treatment.

## Experimental Procedures

### Human plasma samples

This study was reviewed and approved by the local ethics Institutional Review Board (IRB) (Xiangya Hospital, Central South University, IRB-201512526). All experiments were conducted in accordance with the principles of the Declaration of Helsinki. We collected plasma from 90 individuals (45 psoriasis vulgaris patients and 45 sex-, age-, and BMI-matched healthy controls). The subjects' demographic characteristics are summarized in **Table 1**. Inclusion criteria included newly diagnosed and untreated psoriasis patients without any other metabolic comorbidities. Both psoriasis patients and healthy control subjects were older than 18 years of age and gave written informed consent and provided blood samples. Exclusion criteria included the use of subcutaneous and intravenous systemic immunosuppressant medications. Patients were clinically evaluated for psoriasis subtype and PASI score.

### Untargeted metabolomic profiling and data analysis

Metabolite extraction was performed primarily according to previously reported methods 26. In brief, 100 µL samples were extracted by directly adding 300 µL of precooled methanol. After vortexing for 1 min and incubating at -20 °C for 2 h, the samples were centrifuged for 20 min at 4,000 rpm, and the supernatant was transferred to autosampler vials for LC-MS analysis. A quality control (QC) sample was prepared by pooling the same volume of each sample to evaluate the reproducibility of the LC-MS analysis.

The analysis was performed on an ACQUITY UPLC instrument coupled to a Xevo G2-XS QTOF mass spectrometer (Waters, USA). The chromatographic separation of all samples was performed on a BEH C18 column (100 × 2.1 mm, 1.7 µm, Waters, USA), and the column temperature was maintained at 50 °C. The UPLC system was operated with a gradient elution program consisting of water with 0.1% formic acid (mobile phase A) and methanol (mobile phase B). The linear gradient was optimized as follows: 0-2 min, 0% B; 2-12 min, 0-100% B, with a 0.4 mL/min flow rate. Mass spectrometry analysis was conducted in positive and negative ion modes. To ensure m/z accuracy, the m/z values of all ions acquired via the QTOF/MS were real-time adjusted by LockSpray. Leucine-enkephalin was selected as the lock mass compound for positive ion mode ([M + H] + = 556.2771) and negative ion mode ([M - H] - = 554.2615). A QC sample was injected every ten samples to monitor the reproducibility of the analytical platform.

The raw data were imported into Progenesis QI (Waters, USA) software for automatic data processing. The workflow for data processing and analysis included retention time correction, experimental design set-up, peak selection, normalization, deconvolution, and compound identification. For features (m/z extracted with corresponding retention time) extracted by Progenesis QI, metaX 27 was used for statistical analysis, including principal component analysis (PCA), partial least squares discriminant analysis (PLS-DA) and quality control. R^2^ and Q^2^, parameters of PLS-DA, were 0.792 and 0.291, respectively, in the positive mode, with p-value (R^2^) = 0 and p-value (Q^2^) = 0, and in the negative mode, R^2^ = 0.839 and Q^2^ = 0.458, with p-value (R^2^) = 0 and p-value (Q^2^) = 0, respectively.

To identify features detected in samples, the accurate m/z was first derived to match the metabolite from the Metlin 28, BGI library (in house MS/MS library of standards) and HMDB 29 databases. Furthermore, Mummichog 30 was used to leverage the organization of metabolic networks to predict functional activity directly from feature tables, bypassing metabolite identification.

### Mice and treatments

BALB/c mice were purchased from Hunan SLAC Laboratory Animal Co., Ltd. (Hunan, China). The mice were bred and maintained under specific pathogen-free (SPF) conditions and provided with food and water ad libitum. Age-matched and sex-matched mice were used for all experiments in accordance with the National Institutes of Health Guide for the Care and Use of Laboratory Animals. The animal study protocol was approved by the Ethics Committee of Xiangya Hospital (Central South University, China, #2015110134).

BALB/c Mice 6 to 8 weeks of age were treated with daily topical doses of 62.5 mg of 5% IMQ cream (MedShine, cat. 120503, China), which was applied to their shaved backs for 6 consecutive days 31. A scoring system based on the clinical Psoriasis Area and Severity Index (PASI) was used to evaluate the skin inflammation on the skin lesions of mice. Briefly, erythema, scale, and infiltration were each graded on a scale from 0 to 4 as follows: 0, none; 1, slight; 2, moderate; 3, marked; and 4, severe. The level of erythema was scored using a table with red tints. The cumulative score served as a measure of inflammation severity (scale: 0-12) 31. Psoriasis-like inflammation in mice was induced by imiquimod (IMQ) treatment for 3 (n = 6) or 6 (n = 8) consecutive days, and psoriatic mice were compared with untreated normal controls (n = 6). The mouse skin samples were collected and immediately fixed in 4% paraformaldehyde solution (Servicebio, cat. G1101, China) for hematoxylin and eosin (H&E) staining and the epidermal thickness was measured under the microscope. The phenotypes of the psoriatic mice models are shown in **Figure S1**.

### Amino acids and acyl-carnitine quantification

The amino acids and acyl-carnitines in human and mice plasma were measured using ultra-performance liquid chromatography-tandem mass spectrometry (UPLC-MS-MS). Samples were analyzed on a QTRAP 5500 LC-MS/MS system (SCIEX, Framingham, MA) equipped with a Waters UPLC (Waters, USA).

For the quantification of amino acids, 40 μL plasma was mixed with 20 μL stable-isotope-labeled internal standard (IS) in sulfosalicylic acid to precipitate proteins, followed by vortexing and centrifugation (4000 rpm, 4 °C, 20 min). Chromatographic separation was achieved on an HSS T3 column (100 × 2.1 mm, 1.8 µm, Waters, USA), and the column temperature was maintained at 40 °C. The UPLC system employed a gradient elution program consisting of water with 0.1% formic acid (mobile phase A) and acetonitrile (mobile phase B). The linear gradient was optimized and was as follows: 0.3-6 min, 2% to 40% B; 6-9 min, 40% B, 9-9.5 min for 40% to 90% B, with a 0.5 mL/min flow rate. Calibration was achieved by spiking plasma with different quantities of amino acid standards. Data were analyzed using MultiQuant software (SCIEX, USA).

For the quantification of acyl-carnitines, 40 μL plasma was mixed with 10 μL stable-isotope labeled internal standard (IS) in acetonitrile/2-propanol/methanol 3:1:1 (v/v) to precipitate proteins, followed by vortexing and centrifugation (4000 rpm, 4 °C, 20 min). Chromatographic separation was achieved on an HILIC column (100 × 2.1 mm, 1.7 µm, Waters, USA), and column temperature was maintained at 40 °C. The UPLC system employed a gradient elution program consisting of 95% acetonitrile with 10 mM ammonium formate (mobile phase A) and 50% acetonitrile with 10 mM ammonium formate (mobile phase B). The linear gradient was optimized and was as follows: 1-7 min, 95% to 35% B; 7-8 min, 35% B, with a 0.5 mL/min flow rate. Calibration was achieved by spiking plasma with different quantities of amino acid standards. Data were analyzed using MultiQuant software (Sciex, USA).

### L-Carnitine (LC(C0)) supplementation treatment

BALB/c mice 6 to 8 weeks of age were treated with L-carnitine (Sigma-Aldrich, cat. C0158, USA) at a dosage of 1 g/kg/d from day 1 to day 18 by gastric perfusion. IMQ was applied topically to the shaved backs of the mice from day 13 for 6 consecutive days. The mice were imaged and euthanized for skin lesion and spleen analysis on day 19. (The mice were divided into 3 groups: vehicle (IMQ + ig vehicle), LC (IMQ + ig LC), and untreated (Control)).

### Tissue processing

Skin lesions of IMQ-induced mice were cut into small pieces and digested in 5 mL PBS containing 2 mg/mL collagenase type IV (Sigma-Aldrich, cat. V900893, USA) and 1 mg/mL dispase II (Sigma-Aldrich, cat. D4693, USA) while shaking at 37 °C for 90 min. Enzyme activity was stopped using 10% FBS medium. The tissue was further homogenized with a syringe and filtered through a 40 μm cell strainer. The cell strainer was washed with 20 mL PBS followed by centrifugation (500 x g at 4 °C for 10 min). Single-cell suspensions from the spleens were obtained by mashing the spleens through 40 μm cell strainers. The cell strainer was washed with 20 mL PBS followed by centrifugation (500 x g at 4 °C for 5 min), and then red blood cells were lysed using a lysing solution (BD Pharm Lyse™, cat. 555899, USA). Single cells were then stained with fluorescence antibodies for flow cytometry.

### Flow Cytometry

The antibodies are summarized in Table S1. First, Zombie Aqua™ Fixable Viability Dye was used for selecting living cells. Subsequently, Trustain fcX anti-mouse CD16/32 was used to block the Fc receptors of mouse cells. For surface staining, single cells isolated from the skin or spleen were incubated with antibodies at 4 °C for 30 min, followed by washing and centrifugation (500 x g at 4 °C for 5 min). For intracellular cytokine staining (Th1 and Th17), cells were restimulated in 100 μL RPMI supplemented with Golgi-Plug (1:1000, BD, cat. 550529, USA), PMA (50 ng/mL, Applichem, cat. MKCD3841, Germany) and ionomycin (750 ng/mL, Tocris, cat. 56092-81-0, Britain) for 4 to 6 h at 37 °C. After surface staining, cells were permeabilized and fixed in 250 μL BD Cytofix/Cytoperm™ according to the manufacturer's instructions. Then, the cells were washed with permeabilization buffer and stained intracellularly at 4 °C for 30 min in permeabilization buffer. Following surface staining, cells were fixed and permeabilized for intranuclear staining (Tregs), using the eBioscience Foxp3/transcription factor fixation/permeabilization concentrate and diluent from ThermoFisher. The cells were then incubated with anti-mouse/rat Foxp3 antibodies at room temperature for 40 min according to the manufacturer's instructions. Acquisition was performed with FACS Canto II (BD Biosciences). Flow cytometric analysis of live, single cells was performed using FlowJo (Tree Star) software.

### Statistical analysis

All statistical analyses were performed using GraphPad Prism 6 (GraphPad Software, San Diego, CA, USA) or the R (http://www.R-project.org.) with multiple packages, including ggplot2 and dplyr. The significance of differences between groups was determined by 2-tailed unpaired Student's t-test or one-way ANOVA with Dunnett's post hoc test when samples were not distributed normally. All data represent the mean ± SD.

## Results

### Metabolite profiles in psoriasis plasma identified by non-targeted metabolomics

**Figure 1:**
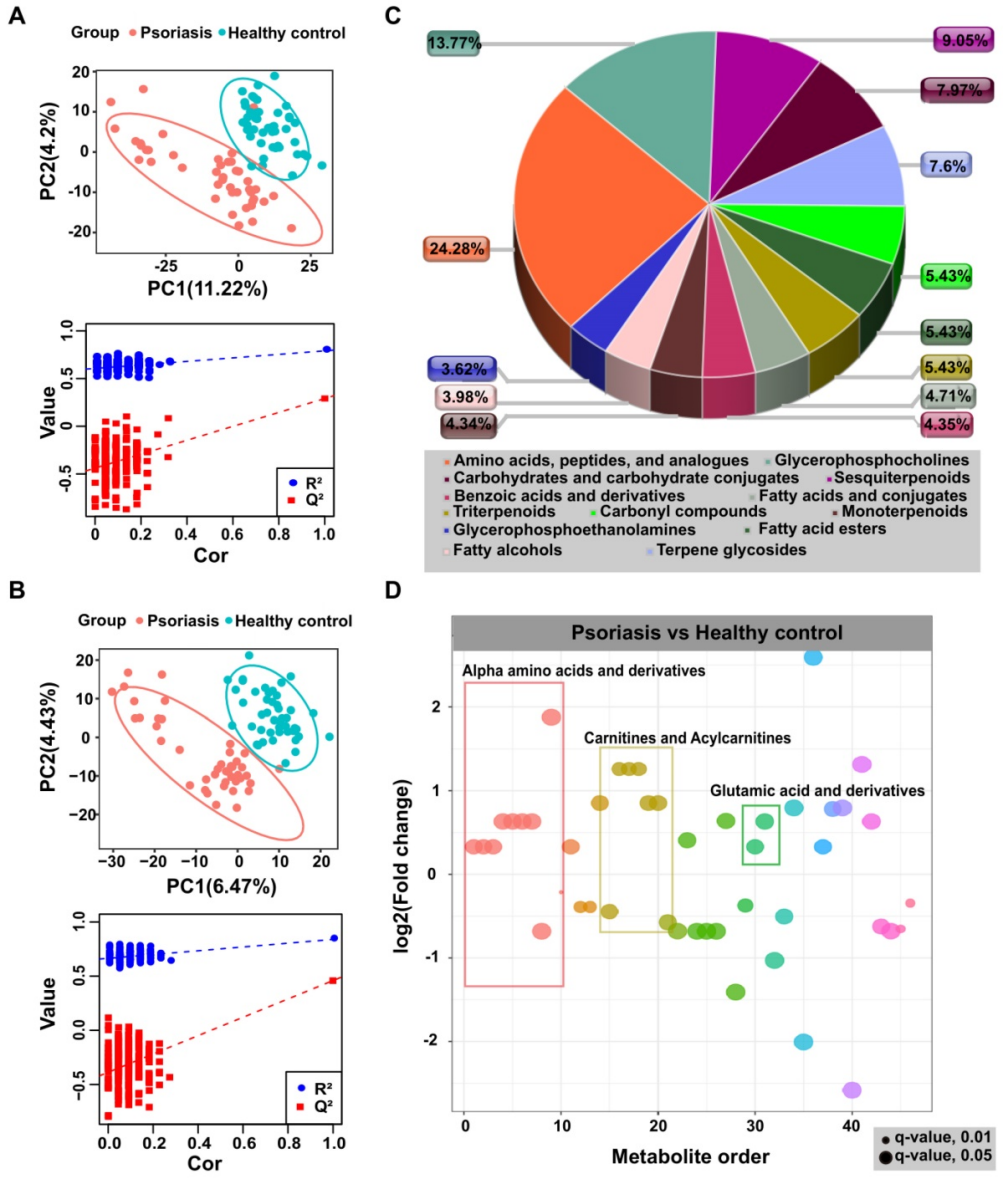
** Non-targeted metabolomics profiling analysis for human plasma.** PLS-DA score plots from the healthy and psoriasis groups in (A) positive mode and (B) negative mode. Validation plots were obtained from 200 permutation tests in (A) positive mode (R^2^ = 0.791, Q^2^ = 0.291) and (B) negative mode (R^2^ = 0.839, Q^2^ = 0.458). (C and D) Metabolomics profiling annotation using database searching and metabolic network analysis to explore the corresponding identification and functional enrichment.

In the non-targeted metabolomic profiling, we detected 5529 and 4289 features in positive and negative modes, respectively. The principal component analysis (PCA) showed no significant difference between the two groups, however, the pooled QC samples clustered together in both positive and negative ion modes **(Figure S2A)**, indicating the authenticity of LC-MS is qualified. Furthermore, the partial least squares discriminate analysis (PLS-DA), a supervised multivariate data analysis method, was conducted to examine differences between psoriasis samples and healthy controls and the PLS-DA model results clearly distinguished psoriasis and healthy control groups in positive and negative modes, as shown in **Figure 1A-B**. Differentially expressed metabolites (**Table S2**) were identified by statistical analysis (VIP ≥ 1 & p.adj < 0.05).

To explore the identities and functional enrichment of the differential features, we searched databases and metabolic networks. As shown in **Figures 1C-D**,** and Figures S1B-C**, the differential features were identified as alpha-amino acids and derivatives, carnitines and acylcarnitines, glutamic acid and derivatives, as well as other compounds. Pathway analysis was performed through MetaboAnalyst 32, which showed that the differentially expressed metabolites in psoriasis patients were enriched in pathways including aminoacyl-tRNA biosynthesis and metabolism of various amino acids, such as alanine, aspartate, glutamate, D-glutamine and D-glutamate, glutathione, arginine, proline, cysteine, and methionine (**Figure S3**). These results were consistent with previous study 33. Furthermore, the metabolic network prediction validated functional enrichment analysis results, revealing that the metabolism in the psoriasis group was centered on histidine and tryptophan metabolism (**Table S2**).

### Amino acid profiles in psoriasis patients and the IMQ-induced psoriasis-like mouse model identified by amino acid-targeted metabolomics

**Figure 2:**
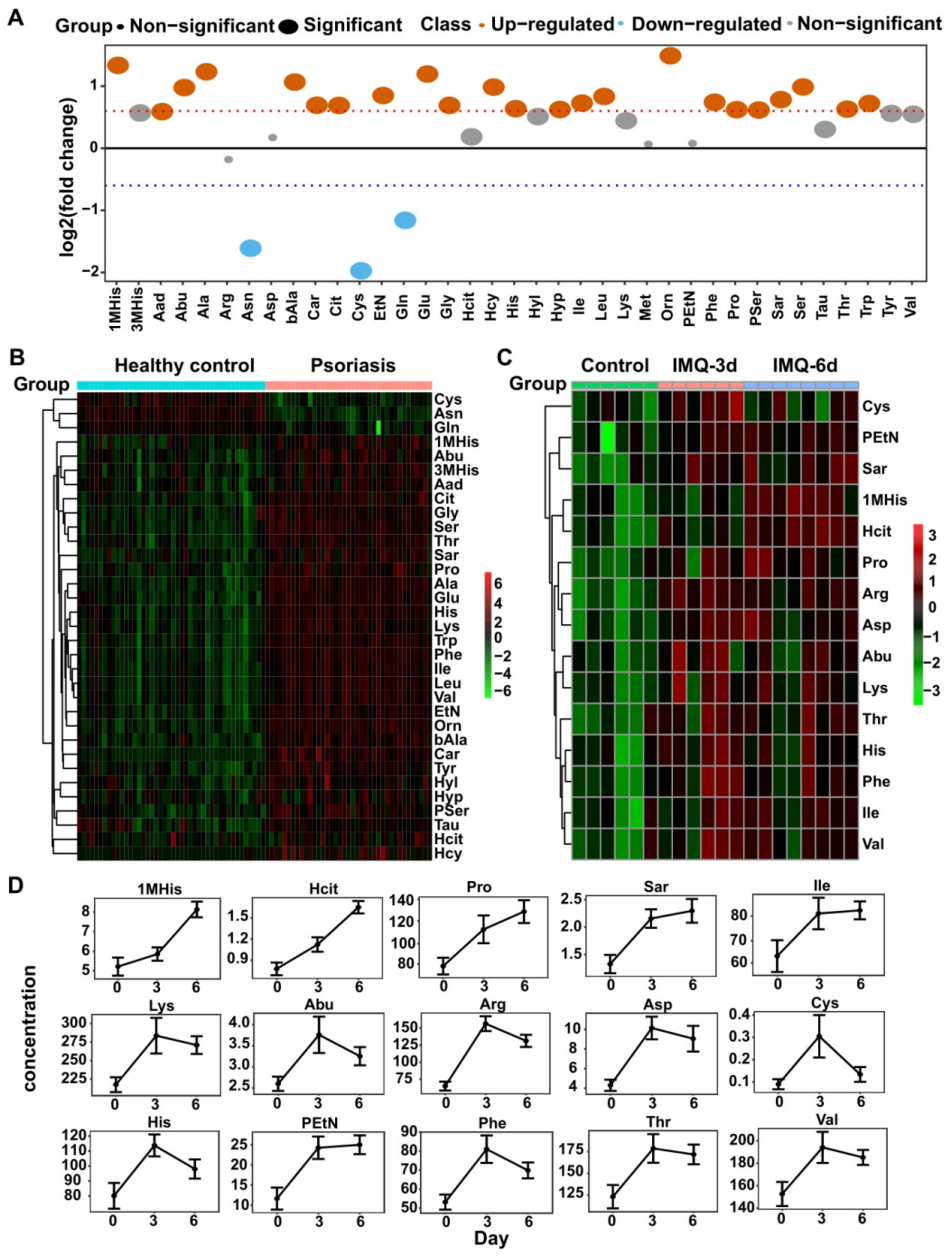
** Amino acid-targeted metabolomics profiling has been examined in human and mice plasma.** (A) The fold change distribution of 37 amino acids in psoriasis versus healthy group. (B) Heatmap of differential amino acids quantified in human plasma. (C) Heatmap of differential amino acids in IMQ-induced mice model at the time points of initial, 3 days, 6 days. (D) The concentration distribution of differential amino acids in IMQ-induced mice model across the three time points.

Our untargeted profiling revealed significant alterations of amino acids in psoriasis patients' plasma, indicating that amino acid metabolism may play an important role in psoriasis pathogenesis. Therefore, we conducted amino acid-targeted metabolomics and identified 37 differentially expressed amino acids (**Figure S4A**). Among these, 26 had greater than 1.5-fold changes in psoriasis patients than control subjects, including 23 upregulated and 3 downregulated amino acids (**Figure 2A-B**).

IMQ, a ligand of TLR-7/8, induces psoriasis-like dermatitis in mouse mimicking human psoriasis pathogenesis and systemic inflammation, including elevated levels of IL-23/IL-17A-Th17 axis pro-inflammatory cytokines and chemokines 34-36. We analyzed the amino acid profiles in the IMQ-induced psoriasis-like dermatitis mouse model by amino acid-targeted metabolomics. As shown in **Figure 2C-D**, we found 15 amino acids with increased levels after treatment with IMQ, largely consistent with the results observed in human psoriasis patients. Among these 15 amino acids, 1-methylhistidine, homocitrulline, proline, sarcosine, isoleucine, and phosphoethanolamine continued to increase after 3 and 6 consecutive days of treatment, while alpha-amino-n-butyric acid, arginine, aspartic acid, cystine, histidine, lysine, phenylalanine, threonine, and valine were increased after treatment for 3 days but declined at day 6. However, their concentrations at 6 days were higher than the corresponding levels in the control group (**Figure 2D**). Also, 37 amino acids with significant differences in the plasma of psoriasis-like dermatitis mice treated with IMQ for 3 or 6 consecutive days were quantified (**Figure S4B**).

**Figure 3:**
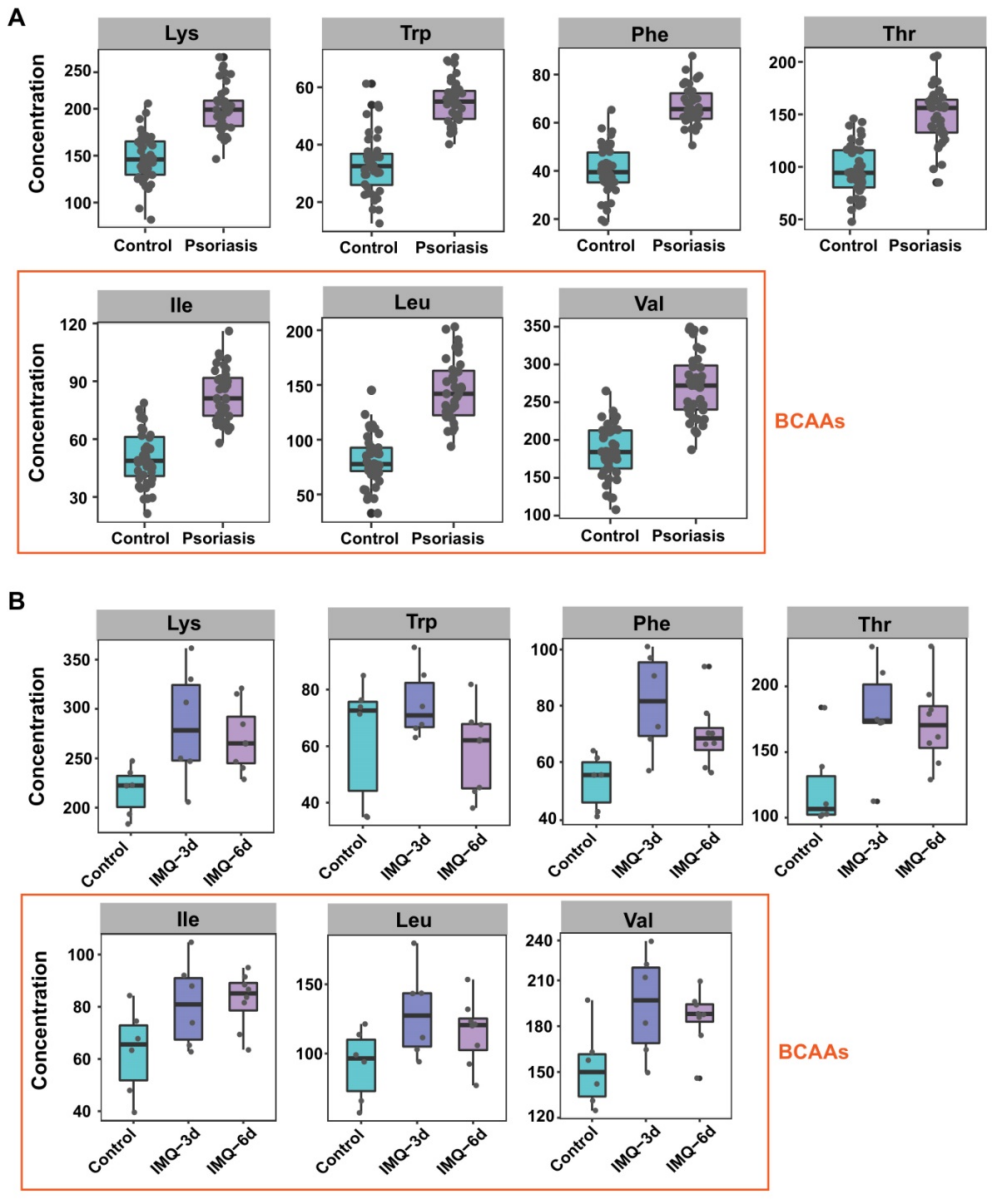
** Seven amino acids are significantly altered in both human and mice plasma.** Boxplot of the 7 differential amino acids including lysine, tryptophan, phenylalanine, threonine and BCAAs (isoleucine, leucine, and valine) in both human (A) and mice (B) plasma.

The amino acids with a significant difference in both psoriasis patients and IMQ-induced psoriasis-like dermatitis mouse model included glutamate, aspartic acid, EAAs (**Figure 3A-B**), BCAAs (isoleucine, leucine, and valine) (**Figure 3A-B**), phosphoserine (Pser), ornithine, other hydrophobic amino acids, including alanine and proline, and the aromatic amino acids (AAAs, phenylalanine and tyrosine). Interestingly, these amino acids are involved in metabolic diseases, including obesity and insulin resistance, which are associated with psoriasis 37-39.

### Carnitine-targeted metabolomics in psoriasis patients and IMQ-induced psoriasis-like dermatitis mouse model

**Figure 4:**
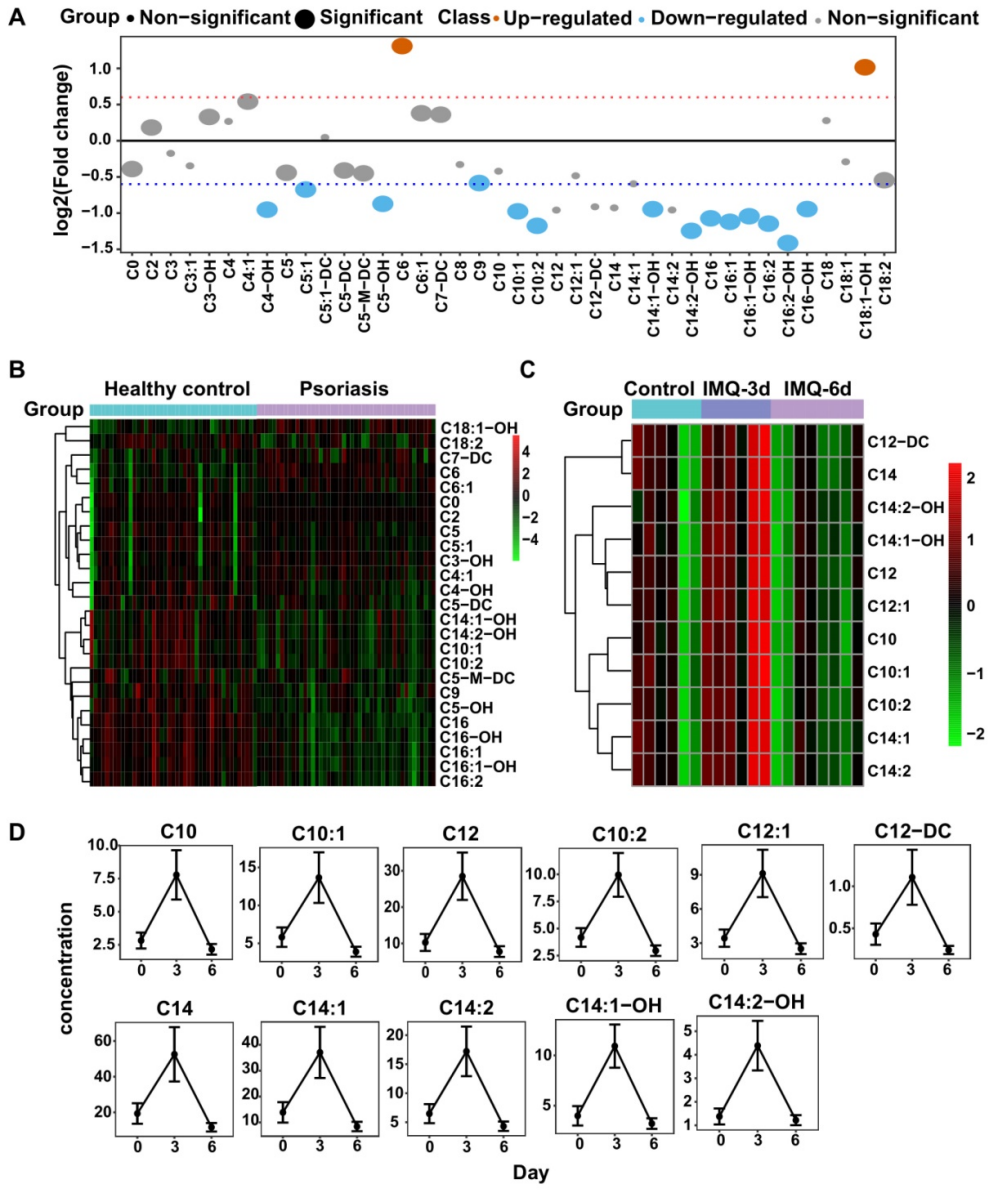
** Acyl-carnitines are quantified in both humans and mice plasma.** (A) The fold change distribution of Acyl-carnitines in psoriasis versus healthy group. (B) Heatmap of differential Acyl-carnitines quantified in human plasma. (C) Heatmap of differential Acyl-carnitines in IMQ-induced mice model at the time points of initial, 3 days, 6 days. (D) The concentration distribution of differential Acyl-carnitines in IMQ-induced mice model across the three time points.

The untargeted profiling showed that free carnitine (C0) and butyrylcarnitine (C4) levels were markedly decreased in psoriasis patients, and the fatty acid beta-oxidation pathway was important in psoriasis pathogenesis. Since carnitine is an essential energy substance that participates in fatty acid beta-oxidation, we performed carnitine-targeted metabolomics to investigate the role of acylcarnitines in psoriasis and identified 40 acylcarnitines with significant differences (**Figure S5A**). Among these acylcarnitines, 16 had greater than 1.5-fold changes in psoriasis patients relative to healthy control subjects, comprising 14 downregulated and 2 upregulated acylcarnitines (**Figure 4A-B**).

In the IMQ-induced psoriasis-like mouse model, we identified 34 acylcarnitines (**Figure S5B**), 11 of which were differentially expressed after IMQ treatment (t-test, p<0.05), including C10, C12, and C14 species. The psoriatic mice treated with IMQ for 3 consecutive days had higher levels of these acylcarnitines than controls, while those treated for 6 days had lower levels of acylcarnitines than normal controls (**Figure 4C-D**). These findings suggested that metabolic perturbations of carnitine analogs to those in psoriasis patients exist in mice with psoriasis induced by IMQ.

### Correlation of identified plasma metabolites with psoriasis severity

**Figure 5:**
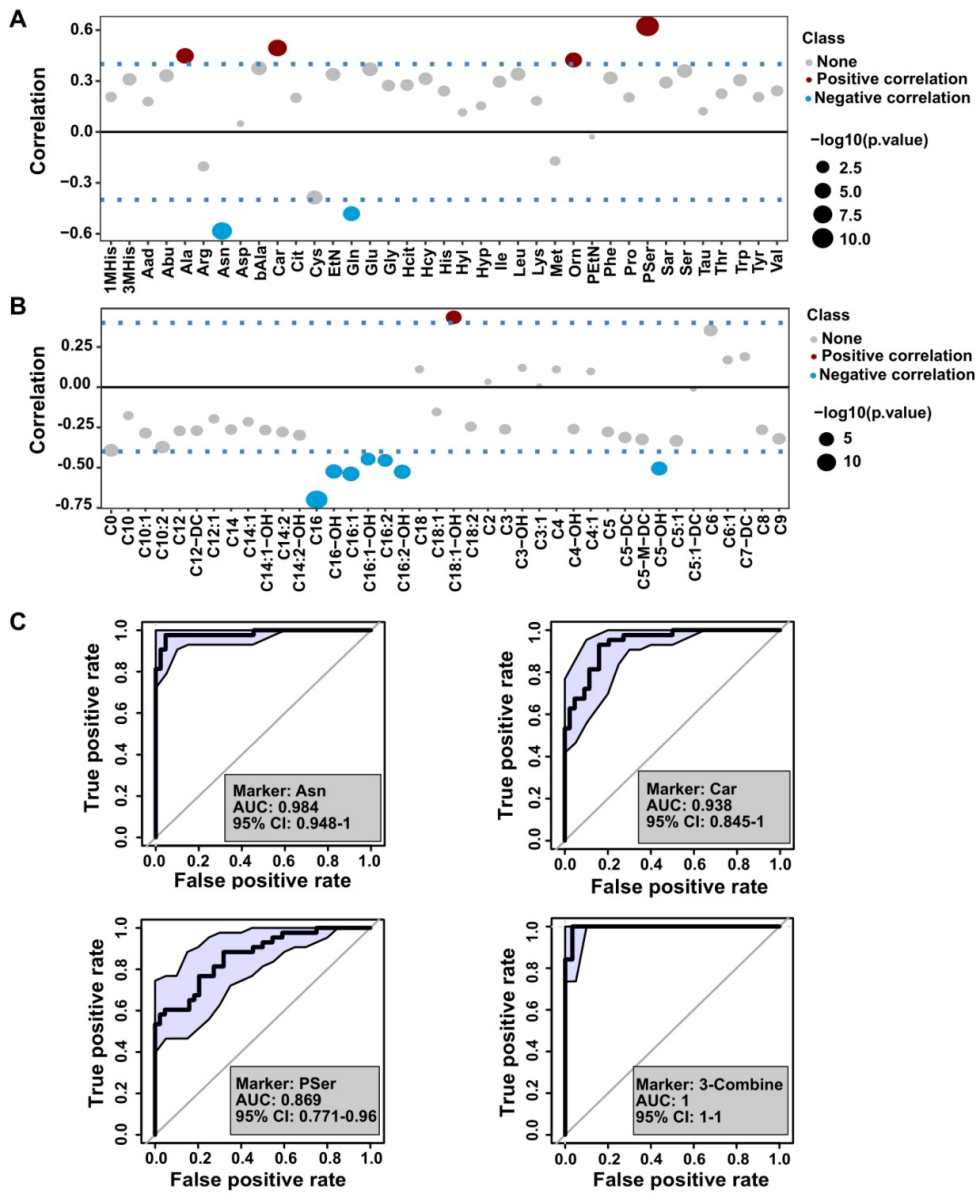
** Biomarker analysis of the amino acids and acyl-carnitines with the severity of psoriasis.** (A and B) The distribution of Pearson correlation coefficients between PASI score and amino acids or acyl-carnitine. (C) Receiver operating characteristic (ROC) curve based on the selected amino acids using single and combination analysis.

The severity of psoriasis has been evaluated by PASI score, therefore, we explored the association between identified metabolites and psoriasis severity by dividing the psoriasis cohort into mild (PASI ≤ 10, n = 25) and moderate-severe (PASI > 10, n = 20) groups. Pearson correlation analysis of PASI scores and the levels of amino acids or acylcarnitines are presented in **Figure 5A-B**, and **Table S3**. We found that glutamine, asparagine, and C16 levels were negatively correlated with the PASI score, whereas there was a positive correlation with the levels of alanine, carnosine, ornithine, and phosphoserine (Pser) (**Figure 5A-B**). These data underscored the potentially beneficial effects of glutamine, asparagine, and C16 on psoriasis vulgaris, providing a novel insight into disease severity assessment, diagnosis, and identifying potential therapeutic targets in psoriasis.

Next, we used multivariate receiver operating characteristic (ROC) curve analysis to discriminate between several classes of amino acids, carnitine, and acylcarnitines. As shown in **Figure 5C and Figure S6**, the combination of asparagine, carnosine, and Pser were correlated with PASI, which could identify psoriasis with an AUC above 0.98.

### Significant attenuation of IMQ-induced psoriasis-like dermatitis by L-Carnitine supplementation

Our results revealed that the concentrations of LC(C0) and most of the acylcarnitine species were significantly decreased in psoriasis patients' plasma relative to control subjects (**Figure 4A-B**). These metabolites were also decreased in mice in which psoriasis was induced by topical application of IMQ for 6 consecutive days relative to control mice (**Figure 4C-D**). These observations were consistent with the possibility that LC(C0) may have potential therapeutic use in psoriasis.

**Figure 6:**
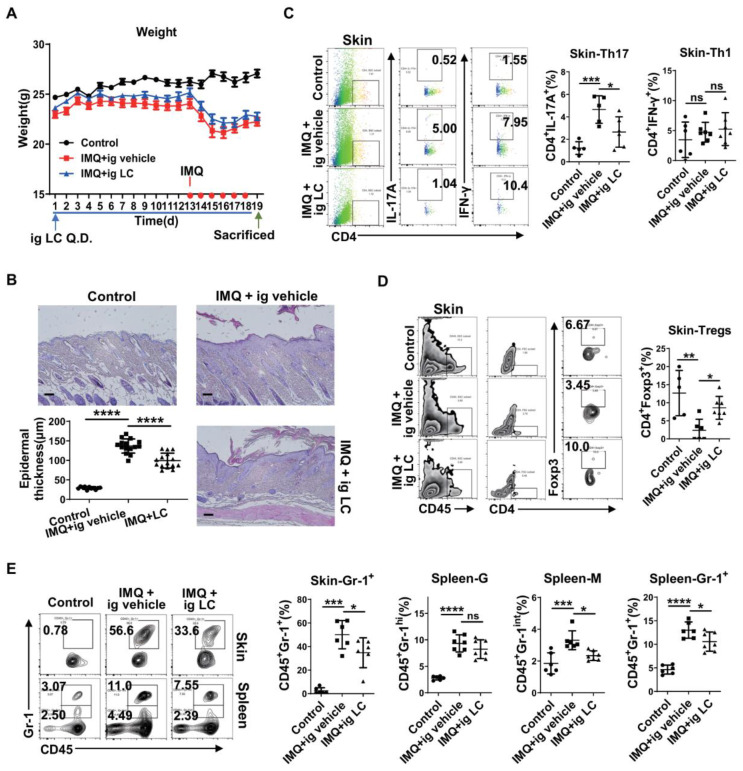
** L-Carnitine supplementation significantly attenuates IMQ-induced epidermal thickening through down-regulating Th17 cells and infiltrating Gr-1^+^ inflammatory cells.** (A) Daily weight record and the specific drug use scheme. (B) The H&E staining of the back skin derived from mice i.g. vehicle (IMQ + vehicle) or L-carnitine (IMQ + LC) or untreated (Control). (One representative mouse from each group is presented, n = 5-7 mice per group). Scale bars: 100 μm. Statistical analysis data is shown in the left-lower panel. Representative flow cytometry panels for quantification of Th1 and Th17 cells (C), Treg cells (D) in skin lesions of BALB/c mice (n = 5-7 mice per group). Statistical analysis data is shown in the right panel. (E) Representative flow cytometry panels for quantification of Gr-1^+^ inflammatory cells in spleen and skin lesions of BALB/c mice (n = 5-7 mice per group). Gr-1^+^ cells were selected from CD45^+^ cells. Statistical analysis data is shown in the right panel. *P < 0.05, **P < 0.01, ***P < 0.001, ****P < 0.0001, ns, not significant. One-way ANOVA with Dunnett's post hoc test was used.

To test the hypothesis that LC(C0) supplementation modulates psoriasis, we performed an intervention study in mice. We treated IMQ-induced psoriatic mice with LC(C0) to study its metabolism. The scheme of drug use and daily weight records are displayed in **Figure 6A**. As expected, compared with vehicle, LC(C0) supplementation markedly decreased IMQ-induced epidermal thickening on day 19 after topical IMQ application for 6 consecutive days (**Figure 6B**). Flow cytometry analysis showed that LC(C0) supplementation markedly decreased IMQ-mediated infiltration of Th17 cells in skin lesions but did not affect Th1 cells (**Figure 6C**). The infiltration of Treg cells in skin lesions was also increased following LC(C0) supplementation (**Figure 6D**). Furthermore, LC(C0) supplementation significantly reduced IMQ-induced accumulation of infiltrating Gr-1^+^ inflammatory cells in skin lesions and spleen, especially that of the monocytic type of Gr-1^+^ inflammatory cells, without affecting the granulocytic type (**Figure 6E**). Our results identified an anti-inflammatory role of LC(C0), which could alleviate IMQ-induced psoriatic inflammation.

## Discussion

Recent studies revealed that metabolic disorders, including abdominal obesity and serum dyslipidemia, were positively correlated with psoriasis risk 9, 10 due to their “low-grade chronic inflammatory state” involving the IL-23/Th17 axis 6. Psoriasis has been identified as a risk factor for metabolic diseases 6, and increasing evidence provides support for this epidemiological observation. For example, increased IL-23 and IL-17 levels were found in diabetes, suggesting their synergistic role in β-cell damage 7. Furthermore, in obesity, elevated concentrations of IL-23 and a Th17 subset expansion in adipose tissue have been observed 8. Thus, there may be shared metabolic pathways of pathogenesis between psoriasis and metabolic disorders. Our study focused on the significantly altered amino acids and carnitine metabolism in psoriasis in both humans and mice.

Perturbations of amino acid metabolism are widespread in metabolic disorders and psoriasis. BCAAs are a group of EAAs comprising leucine, isoleucine, and valine. EAAs, which have rarely been investigated in psoriasis, can only be obtained through the diet and cannot be synthesized in the body from other metabolites 40. Elevated BCAA levels have been widely observed in humans with obesity-associated conditions 37, Consistent with the observations in metabolic diseases, our results revealed increased circulating BCAAs in the plasma of both psoriasis patients and IMQ-treated psoriatic mice (**Figure 3**). We also found that the EEA levels were markedly elevated in both humans and mice (**Figure 3**). Thus, metabolic disorders may be risk factors for the development of psoriasis.

It is not clear whether circulating BCAAs can serve as biomarkers of metabolic disorders and/or are causal agents of disorders. Increasing evidence has indicated the pro-inflammatory roles of BCAAs; for example, BCAAs promote endothelial dysfunction through NF-κB-mediated inflammation 41. BCAAs, especially leucine, are activators of mTOR 42, and can promote oxidative stress and inflammation via mTORC1 activation 43. Moreover, BCAA supplementation suppresses Akt2 activation through mTORC1- and mTORC2-dependent pathways 44, and NF-κB and mTOR are therapeutic targets of psoriasis. The significantly elevated levels of BCAAs in psoriasis observed in the present study suggest that these amino acids may play important roles in the pathogenesis of psoriasis, a possibility that requires further investigation. Interestingly, there is evidence that targeting BCAA catabolism can be used as a strategy to treat obesity-associated insulin resistance by using inhibitors to restore BCAA catabolic flux or reduce protein (BCAA) intake 45.

The foods most enriched in BCAAs are meat, fish, eggs, and dairy products; thus, it might be beneficial for psoriasis patients to limit their intake of these high protein diets. It was reported that psoriasis patients often show unbalanced dietary habits such as higher intake of red meat and fat but lower intake of dietary fibers 46, 47, exacerbating psoriasis via the activation of the nucleotide-binding domain, leucine-rich repeat-containing family, TNF-α/IL-23/IL-17 pathway and reactive oxygen species 48, 49. Therefore, nutritional strategies might be beneficial to relieve psoriatic symptoms and severity.

Glutamine, an immunomodulatory nutrient, is the most abundant free amino acid in plasma and has important metabolic roles in providing intermediates to the tricarboxylic acid cycle (TCA cycle) 50, 51. Highly proliferating cells, such as cancer cells, are particularly dependent on glutamine for biosynthesis 52. Furthermore, total body glutamine deficiency has been observed in catabolic states such as trauma, infection, and sepsis 53. Consistent with a previous study 54, we found significantly decreased glutamine levels and markedly increased glutamate levels in psoriasis patients' plasma (**Figure 2**). These findings support the hypothesis that glutamine-cycling pathways are prominently involved in the development of psoriasis vulgaris. Furthermore, a strong inverse correlation has been reported between glutamine levels and BCAAs in glutamine- and glutamate-fed mice 39, revealing distinct roles of glutamine versus BCAAs that were also highlighted in the Framingham Heart Study cohort 39. These observations underscored the potentially beneficial effects of glutamine on cardiometabolic risk and showed that glutamate might confer an adverse metabolic risk 39. It was reported that parenteral glutamine supplementation in the intensive care setting reduced the morbidity and mortality rate 53. More importantly, in psoriasis, we found that the glutamine level was negatively correlated with the PASI score (**Figure 5A**), strongly supporting our hypothesis that glutamine supplementation or the inhibition of glutamate accumulation is beneficial for psoriasis patients, at least for patients comorbid with metabolic diseases.

Besides conducting metabolic profiling in psoriasis patients, we investigated, for the first time, metabolic changes of amino acids and carnitines in the IMQ-induced psoriatic inflammation. A comparison of the metabolomics of psoriasis patients with those of mice with IMQ-induced psoriatic inflammation suggested that amino acids and carnitines modulate the process of psoriasis and/or serve as biomarkers in psoriasis, a possibility that needs further investigation. Interestingly, we found that LC(C0) supplementation significantly attenuated IMQ-induced psoriasis-like inflammation in mice (**Figure 6**), revealing its anti-inflammatory role in psoriasis.

Deficiencies in various circulating carnitine and acylcarnitines in psoriasis patients, as observed in the present study (**Figure 4A-B**), can also be explained by the increase in fatty acid oxidation in skin lesions and the increased activity of carnitine palmitoyltransferase-1 (CPT-1) 55 due to the rapidly elevated energy consumption of proliferating cells. CPT-1 is the key enzyme responsible for the rate of long-chain fatty acid oxidation in mitochondria, and it has been reported that inhibiting CPT-1 activity with etomoxir alleviates psoriatic inflammation 55. Consistent with these observations, we found that C16 species levels, including C16, C16-OH, C16:1, C16:1-OH, C16:2, and C16:2-OH, were reduced in psoriasis patients (**Figure 4A-B**). More importantly, we found a negative correlation between C16 and the PASI Score (**Figure 5B**), identifying C16 as a potential protective marker in psoriasis severity assessment. Furthermore, carnitine-acylcarnitine translocase (CACT) and carnitine palmitoyltransferase-2 (CPT-2), located on the inner mitochondrial membrane, might also be promising therapeutic targets for preventing the translocation and transformation of acylcarnitines, respectively.

## Conclusion

Our study demonstrates the potential value of integrating clinical, experimental, and metabolomic data from psoriasis vulgaris patients and mice with IMQ-induced psoriasis and conducting detailed phenotyping. Our results suggest that the amino acids, especially EAAs and BCAAs, and carnitine metabolism is significantly altered in psoriasis and may play key roles in the pathogenesis of psoriasis. Further studies are needed to elucidate whether elevated levels of EAAs, BCAAs, and glutamate, downregulated glutamine, and significantly reduced carnitine and acylcarnitine levels can predict psoriasis risk and to explore the underlying mechanisms.

## Supplementary Material

Supplementary figures and tables.

## Figures and Tables

**Table 1:** Demographics of psoriasis patients and healthy control subjects

Characteristics	Psoriasis Patients	Healthy Control subjects	P-value
Number	45	45	1
Age in years	40.64±12.00	39.42±8.95	0.37
Gender	25 males, 20 females	24 males, 21 females	1
BMI	22.03±3.20	22.38±4.01	0.97
Race/ethnicity	100% Chinese	100% Chinese	1
PASI score	10.11±7.46	N/A	<0.001

Abbreviations: N/A, not applicable; PASI, Psoriasis Area and Severity Index; BMI, Body Mass Index. Values are presented as the means ± standard deviation. P-value was calculated by unpaired-Wilcoxon test.

## Abbreviations

IMQ: imiquimod
EAAs: essential amino acids
BCAAs: branched-chain amino acids
C16: palmitoylcarnitine
LC(C0): L-carnitine (free carnitine)
PASI score: the Psoriasis Area and Severity Index score
