# Supplementary material for: Metabolomic profiling reveals amino acid and carnitine alterations as metabolic signatures in psoriasis
Source: Theranostics. 2021 Jan 1;11(2):754–67. doi: 10.7150/thno.51154 (PMC7738860; doi:10.7150/thno.51154)
Supplement: Supplementary file 1 — Supplementary figures and tables. [file thnov11p0754s1.pdf]

Figure S1. The phenotypes of IMQ-induced psoriasis mice models

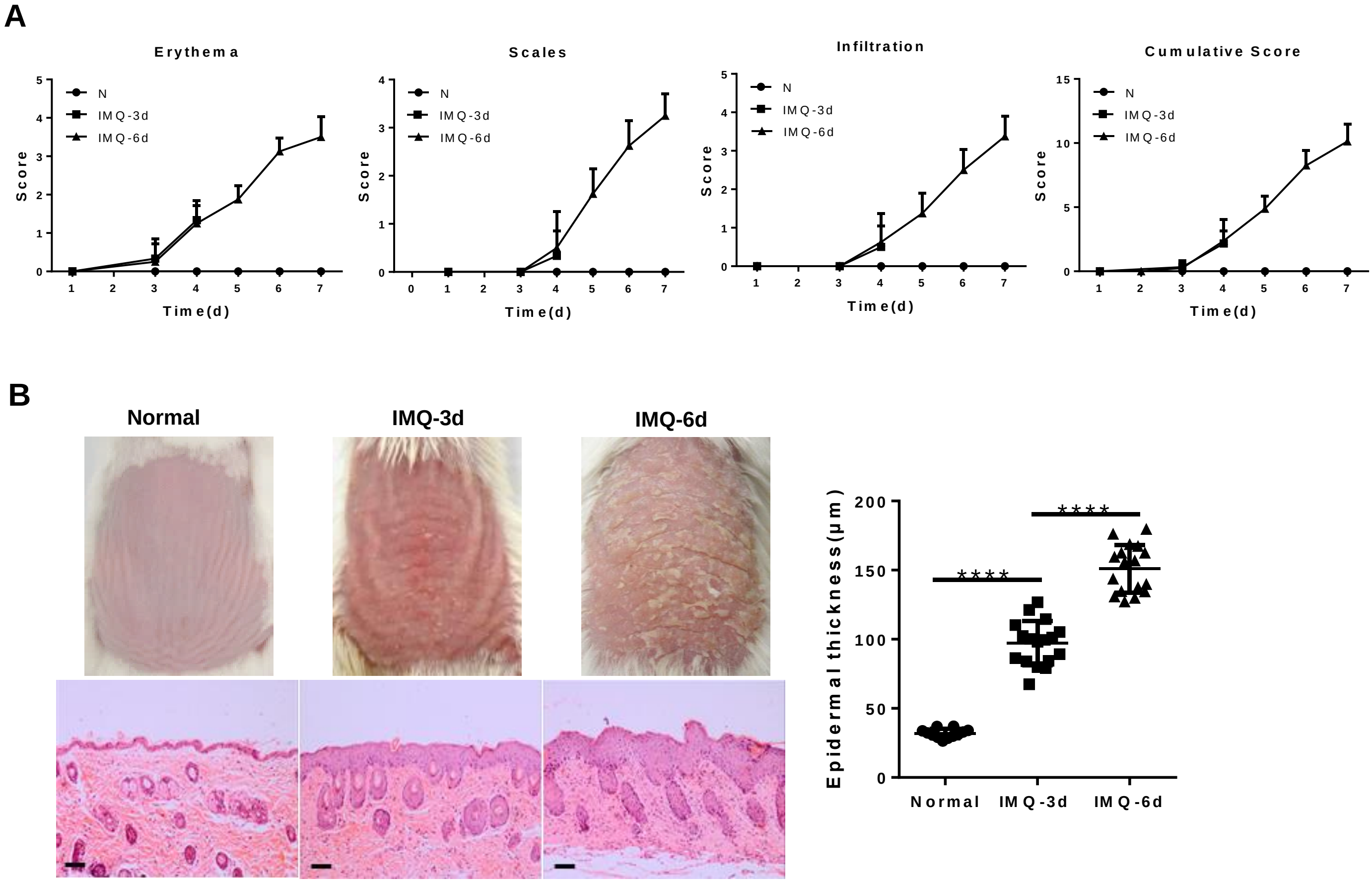

Figure S2. Quality control of Metabolomics profiling and metabolites classes identified in profiling

A

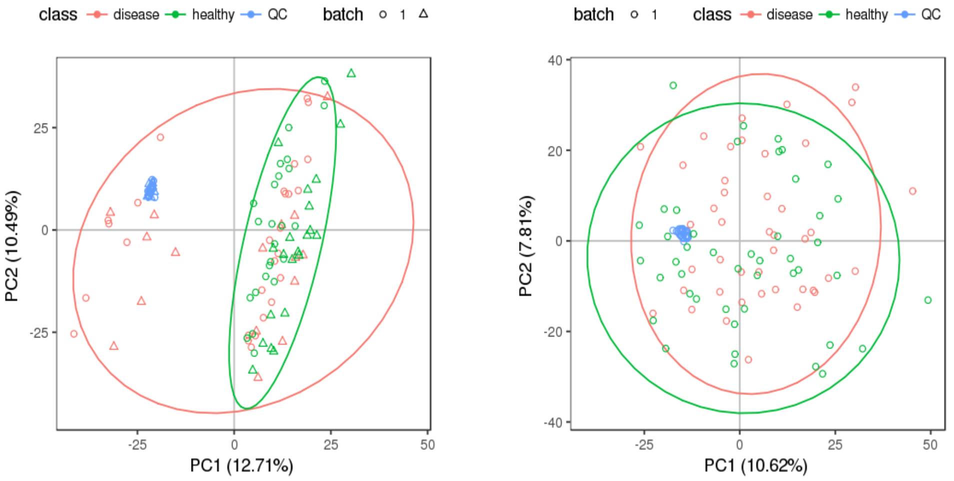

B

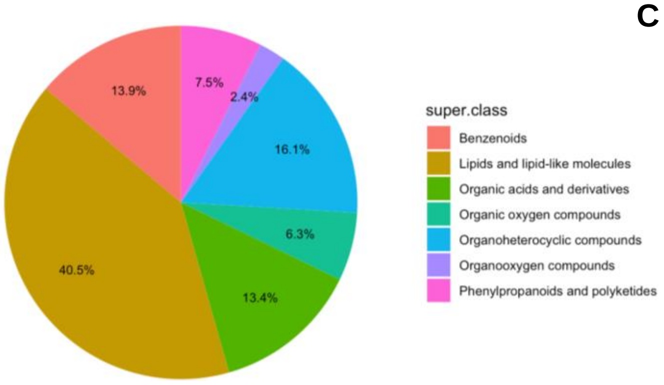

C

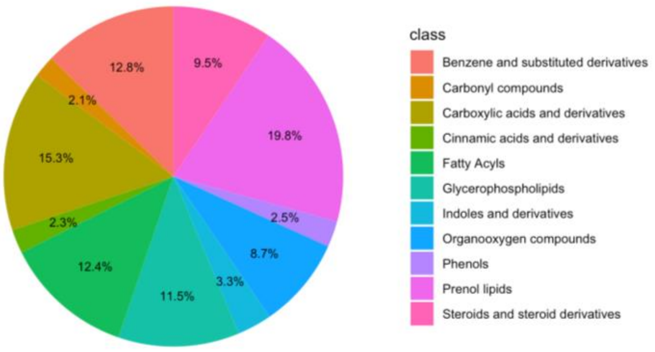

Figure S3. Pathway enrichment using amino acids, acyl-carnitines and identified differential metabolites

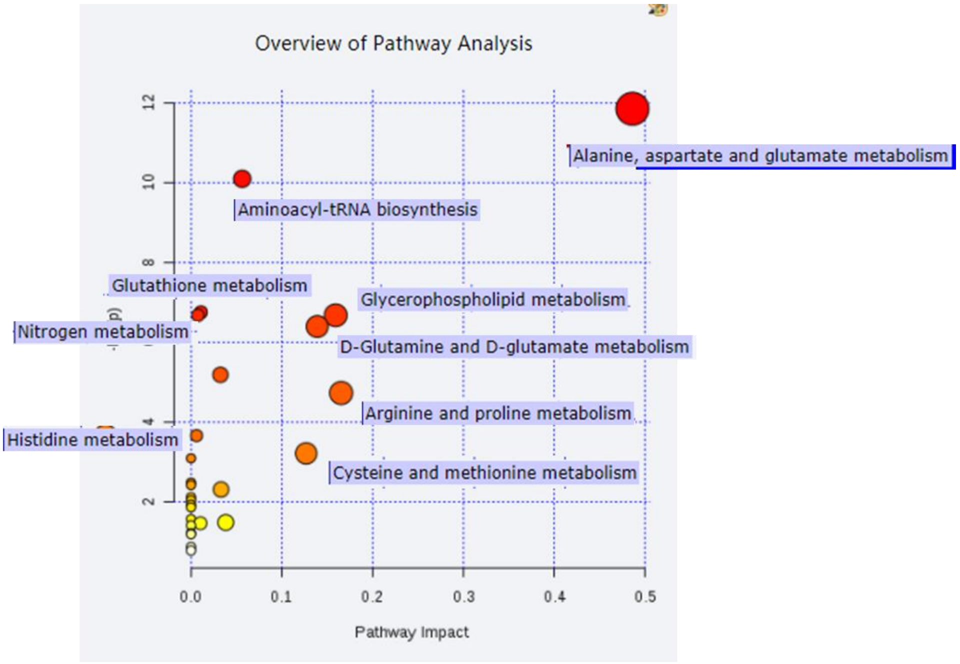

Figure S4. boxplot of amino acids in plasma of humans and mouse models

A

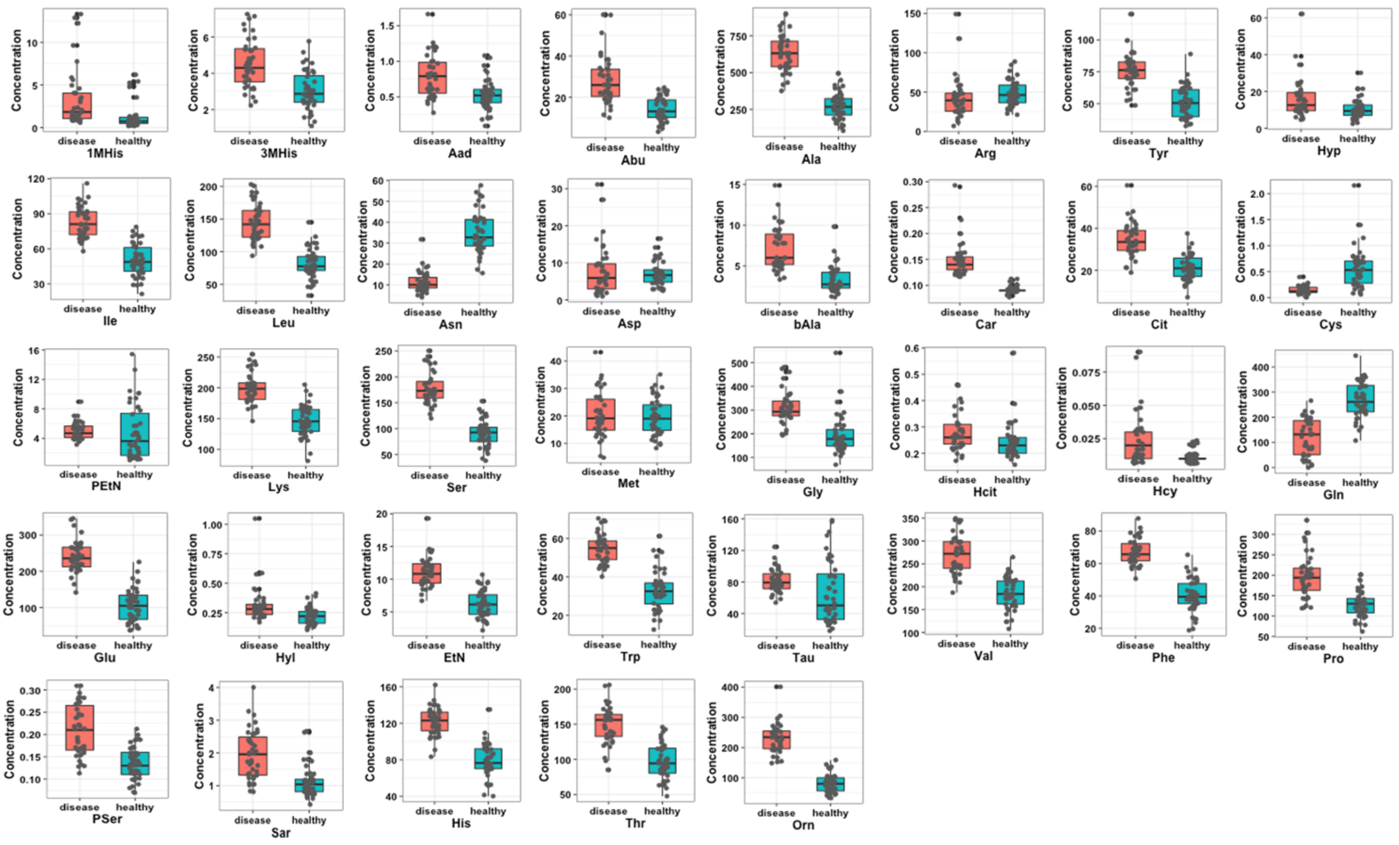

B

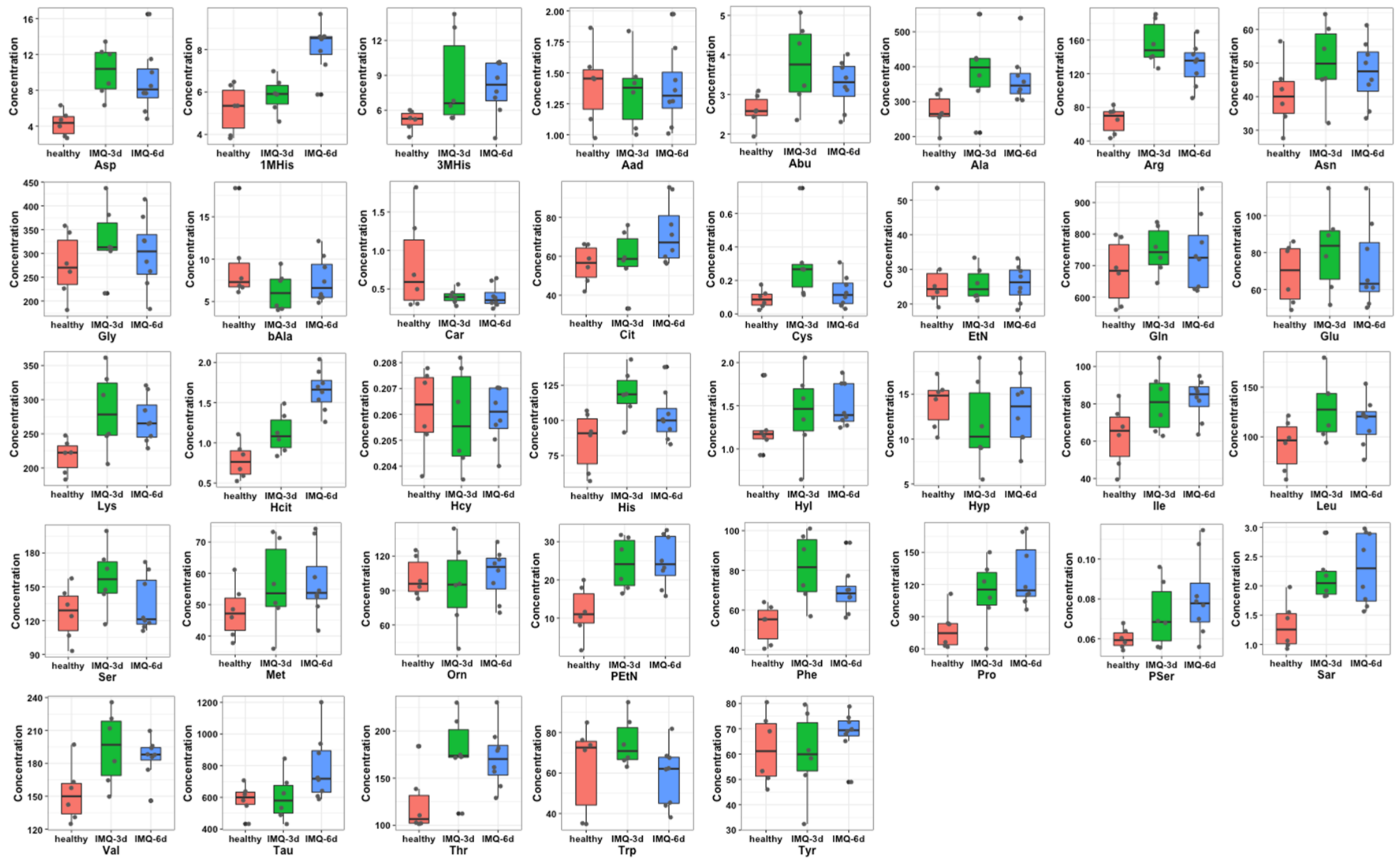

Figure S5. boxplot of carnitines in plasma of humans and mouse models

A

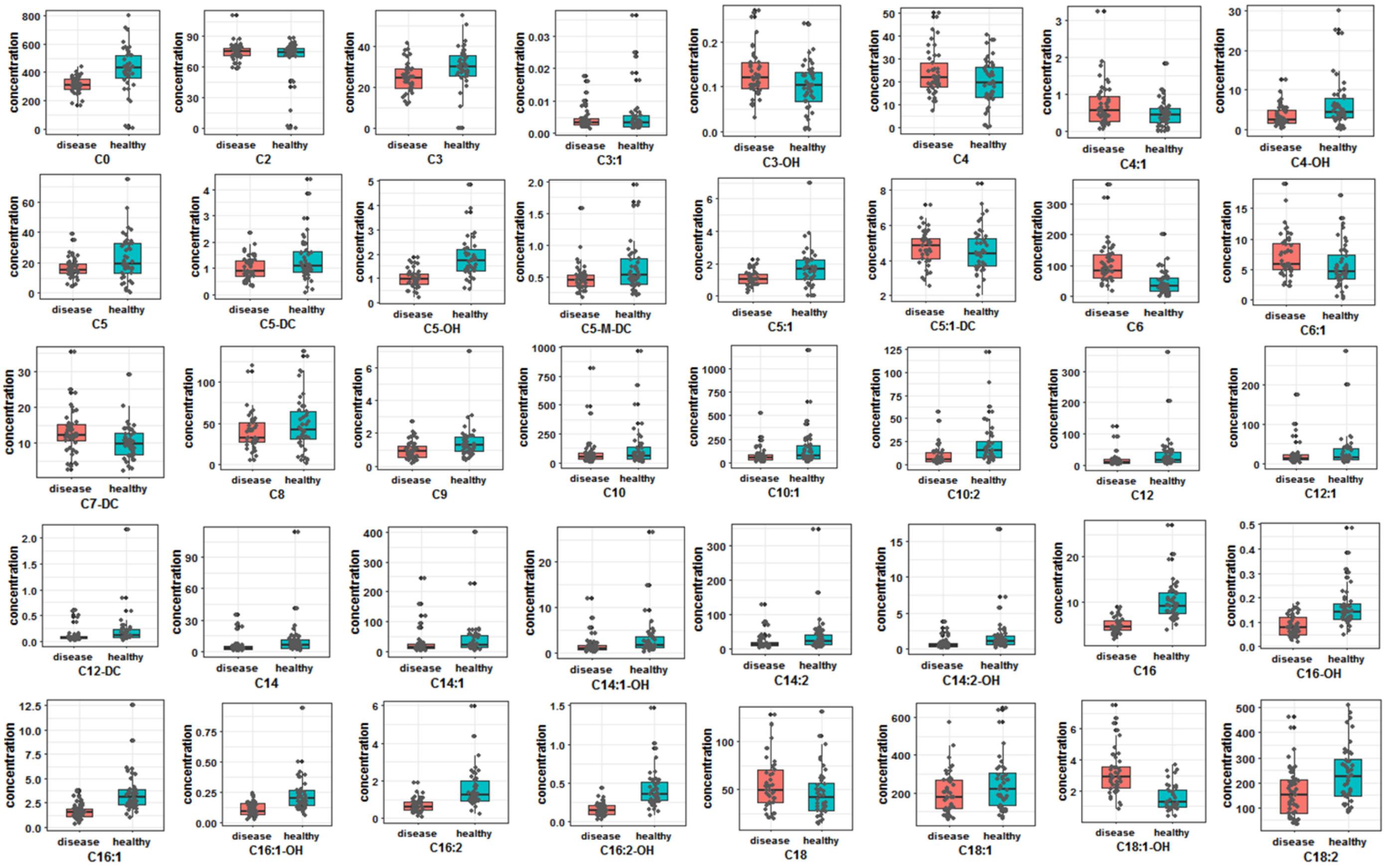

B

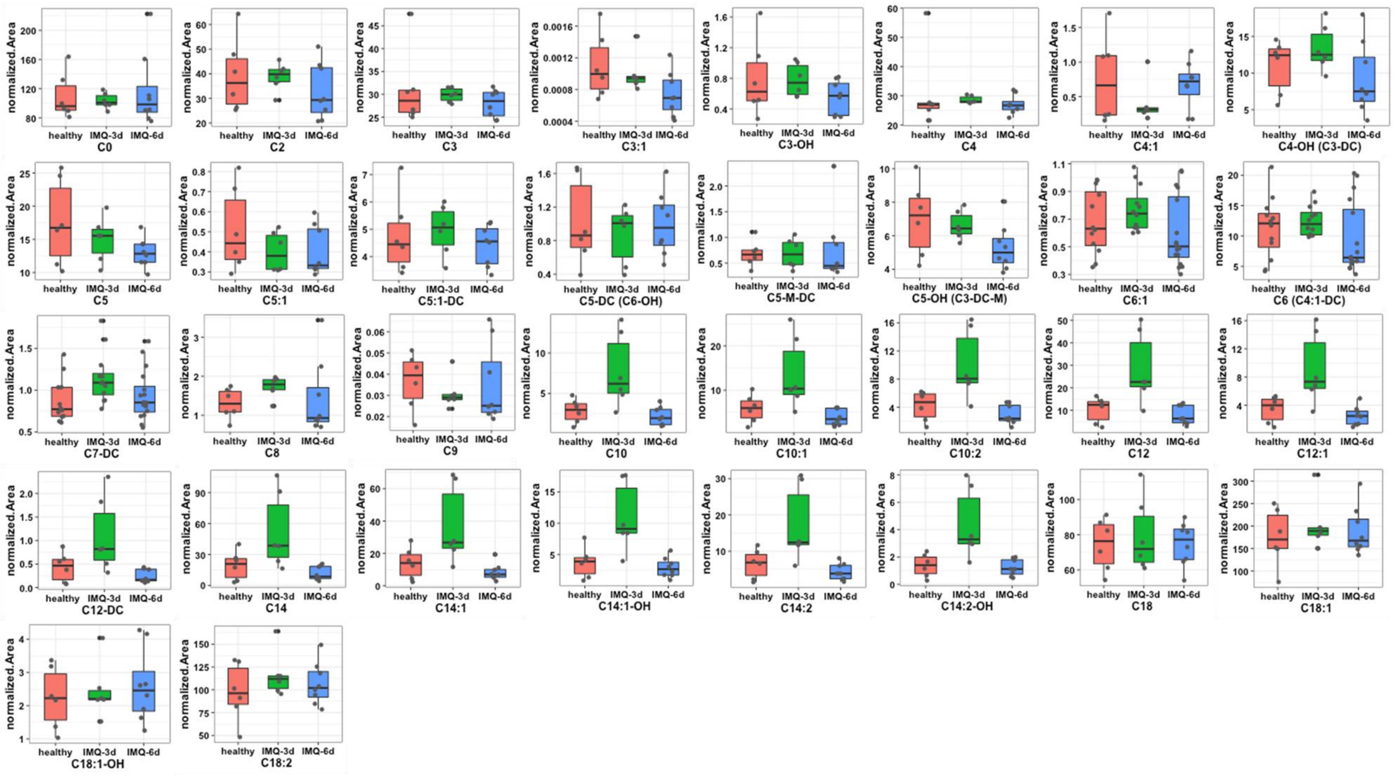

Figure S6.

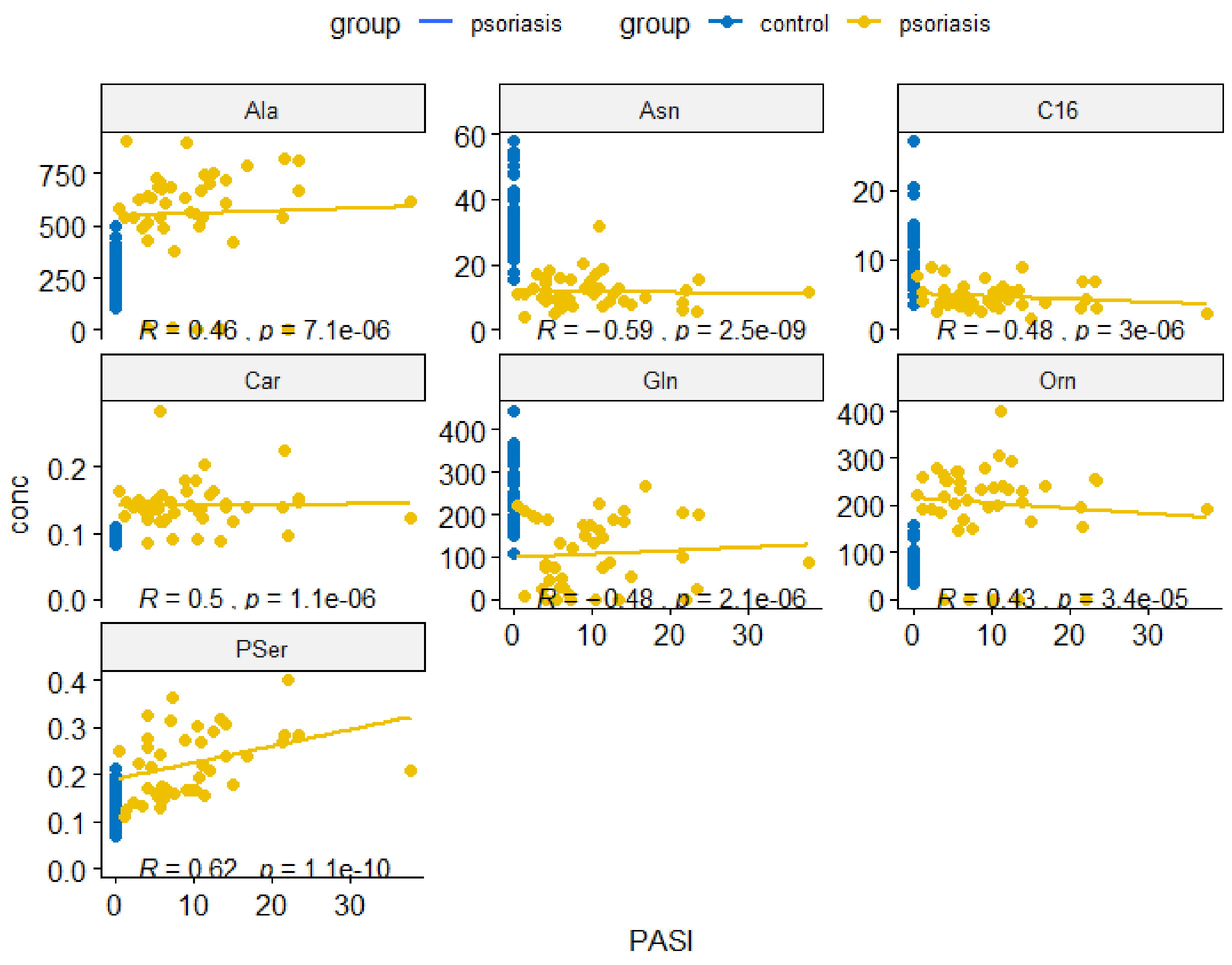
group psoriasis
group control psoriasis
Ala
Asn
C16
Car
Gln
Orn
PSer
R = 0.46 , p = 7.1e-06
R = −0.59 , p = 2.5e-09
R = −0.48 , p = 3e-06
R = 0.5 , p = 1.1e-06
R = −0.48 , p = 2.1e-06
R = 0.43 , p = 3.4e-05
R = 0.62 , p = 1.1e-10
conc
PASI

Table S1. A list of the antibodies for flow cytometry.

For mice	Source
Zombie Aqua™ Fixable Viability Dye	BioLegend
Trustain fcX anti-mouse CD16/32	BioLegend
APC/Cy7 anti-mouse CD45	BioLegend
PerCP/Cy 5.5 anti-mouse Gr-1	BioLegend
PE Rat anti-mouse IL-17A	BD Pharmingen
APC anti-mouse IFN-r	BioLegend
FITC anti-mouse CD4	BioLegend
Anti-mouse CD25 APC	eBioscience
Anti-mouse/rat Foxp3 PE	eBioscience

Table S2. Leverages the organization of metabolic networks to predict functional activity directly from feature tables, bypassing metabolite identification

Mode	Pathway	Pathway total	Hits.total	Hits.sig	p.value
positive	Butanoate metabolism	34	29	4	0.0002
positive	Histidine metabolism	33	16	3	0.0002
positive	Tryptophan metabolism	94	60	5	0.0002
positive	Saturated fatty acids beta-oxidation	36	17	3	0.0002
positive	Glycine, serine, alanine and threonine metabolism	88	41	4	0.0002
positive	Vitamin B3 (nicotinate and nicotinamide) metabolism	28	20	3	0.0002
positive	Valine, leucine and isoleucine degradation	65	43	4	0.0002
positive	Vitamin B2 (riboflavin) metabolism	8	4	2	0.0002
positive	Di-unsaturated fatty acid beta-oxidation	26	6	2	0.0003
positive	Fatty acid activation	74	27	3	0.0003
positive	Lysine metabolism	52	28	3	0.0003
positive	Mono-unsaturated fatty acid beta-oxidation	19	7	2	0.0004
positive	Dimethyl-branched-chain fatty acid mitochondrial beta-oxidation	19	8	2	0.0004
positive	Alanine and Aspartate Metabolism	30	12	2	0.0007
positive	Vitamin B1 (thiamin) metabolism	20	13	2	0.0007
positive	Vitamin B9 (folate) metabolism	33	17	2	0.0011
positive	Propanoate metabolism	31	21	2	0.0016

Table S3. Correlation of amino acids and carnitines with PASI

Component.Name	Correlation	p.value	compoundID	Correlation	p.value
1MHis	0.2065	0.0536	C0	-0.2827	0.0073
3MHis	0.3099	0.0033	C10	-0.0395	0.7134
Aad_2	0.1788	0.0956	C10:1	-0.1239	0.2474
Abu	0.3317	0.0016	C10:2	-0.1944	0.0679
Ala	0.4484	0.0000	C12	-0.1423	0.1834
Arg_2	-0.2031	0.0578	C12-DC	-0.1400	0.1908
Asn	-0.5838	0.0000	C12:1	-0.1053	0.3260
Asp	0.0488	0.6516	C14	-0.1352	0.2066
bAla	0.3755	0.0003	C14:1	-0.1030	0.3366
Car	0.4947	0.0000	C14:1-OH	-0.1863	0.0805
Cit	0.2009	0.0605	C14:2	-0.1405	0.1892
Cys	-0.3862	0.0002	C14:2-OH	-0.1829	0.0862
EtN	0.3388	0.0012	C16	-0.4737	0.0000
Gln	-0.4818	0.0000	C16-OH	-0.3904	0.0002
Glu	0.3695	0.0004	C16:1	-0.3675	0.0004
Gly	0.2734	0.0100	C16:1-OH	-0.3374	0.0012
Hcit	0.2762	0.0092	C16:2	-0.3056	0.0036
Hcy	0.3131	0.0030	C16:2-OH	-0.3942	0.0001
His	0.2412	0.0236	C18	0.1011	0.3457
Hyl_1	0.1150	0.2860	C18:1	-0.1346	0.2087
Hyp	0.1539	0.1524	C18:1-OH	0.3736	0.0003
Ile	0.2959	0.0051	C18:2	-0.1960	0.0656
Leu	0.3404	0.0012	C2	0.0832	0.4382
Lys	0.1830	0.0878	C3	-0.1856	0.0816
Met	-0.1710	0.1111	C3-OH	0.1115	0.2984
Orn	0.4240	0.0000	C3:1	-0.0944	0.3789
PEtN	-0.0293	0.7867	C4	0.0710	0.5086
Phe_2	0.3183	0.0025	C4-OH	-0.2125	0.0456
Pro	0.2034	0.0573	C4:1	0.2046	0.0545
PSer	0.6234	0.0000	C5	-0.1365	0.2022
Sar	0.2914	0.0059	C5-DC	-0.1694	0.1125
Ser	0.3591	0.0006	C5-M-DC	-0.1746	0.1018
Tau	0.1211	0.2610	C5-OH	-0.3493	0.0008
Thr_2	0.2252	0.0349	C5:1	-0.2113	0.0468
Trp	0.3056	0.0038	C5:1-DC	0.0315	0.7697
Tyr_1	0.2057	0.0545	C6	0.3533	0.0007
Val_1	0.2420	0.0231	C6:1	0.2500	0.0181
			C7-DC	0.1063	0.3216
			C8	-0.0839	0.4342
			C9	-0.1719	0.1072
